# Artificial intelligence diagnosis and heatmap agent for mitral valve prolapse using 3D cine echocardiography

**DOI:** 10.1016/j.isci.2025.113033

**Published:** 2025-06-28

**Authors:** Defu Zhang, Miaoru Yu, Xueyuan Lin, Ying Guo, Xiaohua Liu, Qian Liu, Xiaofang Zhong, Yuanyuan Sheng, Shuyu Luo, Yuxiang Huang, Lixin Chen, Jinfeng Xu, Xiaoxuan Lin, Yingying Liu

**Affiliations:** 1 Department of Ultrasound, The Second Clinical Medical College, Jinan University (Shenzhen People's Hospital), Shenzhen 518020, China; 2 Post-doctoral Scientific Research Station of Basic Medicine, Jinan Unviersity, Guangzhou 510632, China

**Keywords:** Health sciences, Biological sciences, Computer science

## Abstract

The detection rate of mitral valve prolapse (MVP) has been increased by using 3D echocardiography. It would be clinically meaningful for accurate assessment of prolapse histology with advanced deep learning algorithms. However, recent studies mainly focus on automatic measurement and reconstruction of cardiac structures using voxel data, while seriously neglecting the automatic diagnosis and visualization using cine data. To address the gap, we propose an artificial intelligence agent to study diagnosis and heatmap for MVP. We include 481 cardiac cycles (8422 frames) from 151 subjects. Our agent achieves the AUC, accuracy and F1 of MVP diagnosis are 99.54%, 95.08%, and 94.12% in patient-levels. The Dice and Iou of heatmap of MVP are 70.35% and 56.39%, which is about 5% higher than that between senior and junior physicians. Our agent would provide standardizing diagnosis and intuitive visualization for the surgical management and treatment plans of MVP.

## Highlights

• An MVP diagnosis and visualization AI agent is developed using 3D cine echocardiography
• The systolic recognition model integrates two sub-networks managed by active-if module
• The saliency segmentation model is supervised by segmentation cue and edge boundary cue
• The boundary cue of saliency segmentation model is guided by edge-belt instead of edge-line

## Graphical abstract

**Figure:**
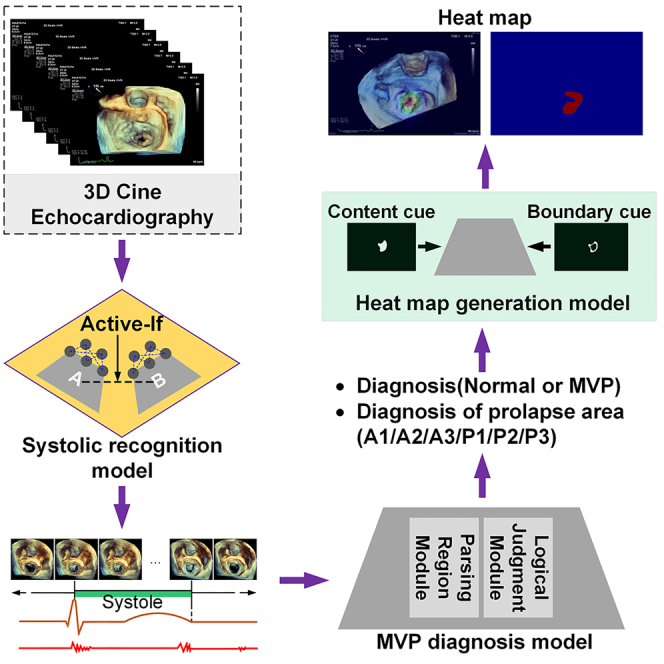

## Introduction

Mitral valve prolapse (MVP) is common valve disease that can lead to mitral regurgitation and even heart failure.^1^^,^^2^ 3D echocardiography is widely used in clinical diagnosis of MVP due to its intuitive 3D structure reconstruction.^3^^,^^4^ Computer aided systems can help to make faster diagnosis and standardize reproducibility by reducing human error. However, recent studies mainly focus on automatic measurement and reconstruction of cardiac structures using voxel echocardiography,^5^^,^^6^^,^^7^^,^^8^^,^^9^ while seriously neglecting the automatic diagnosis and visualization using cine echocardiography. In clinical practice, physicians need to carefully observe cine echocardiography to identify MVP. Due to factors such as artifacts and blurriness, it is easy to misdiagnose, especially for junior physicians. The above situation drives us to develop an artificial intelligence (AI) agent that can achieve automatic diagnosis and visualization of heatmap of MVP.

Fortunately, many models have been proposed for automatic diagnosis of medical imaging. Deep learning structures such as convolutional neural network (CNN)^10^^,^^11^^,^^12^ and Transformer^13^^,^^14^^,^^15^ are widely used as basic feature extraction networks. And the recently proposed base structures such as Mamba and kolmogorov-arnold networks (KAN) have also been successfully applied.^16^^,^^17^^,^^18^^,^^19^^,^^20^ Although these models have strong feature extraction capabilities, they can not achieve best performance in our task. This paper uses a classification model to recognize systolic frames and a segmentation model to segment the anterior leaflet (AL), posterior leaflet (PL) and MVP for systolic frames. The diagnosis of MVP is based on the presence of prolapsed area. The diagnosis of severe zone location of MVP is based on the position relationship among AL, PL and the prolapse area.

For the saliency segmentation of low contrast objects similar to MVP, a series of models have been proposed for polyp^21^^,^^22^^,^^23^^,^^24^^,^^25^^,^^26^^,^^27^^,^^28^ and camouflaged objects.^29^^,^^30^^,^^31^^,^^32^^,^^33^ There are mainly two threads to improve accuracy in these models. One is the coarse-to-fine structure from top to down. Fan^21^ proposed reverse attention to fuse prediction maps from different layers. Dong^22^ proposed similarity aggregation module based on graph convolution to fuse two prediction maps. Wei^23^ proposed shallow attention module with a simple rule function to strengthen foreground and weaken background. Wu^24^ proposed multiscale spatial reverse attention with trainable weights of different scales and parallel convolutional structure. Xiao^25^ used contrastive transformer backbone to obtain the long-range dependence and highly structured feature map space. Ji^26^ utilized progressive grouped convolutional fusion module to extract multi-scale features. Cao^27^ employed weighted dual-branch feature fusion network which includes scale progressive feature fusion and scale-aware feature fusion. Xu^28^ integrated frequency domain into multi-scale alignment.

The other thread is boundary guidance. Jin^29^ utilized the first two layers of features to extract boundary and the last three layers of features to extract contents. Cai^30^ developed a boundary-aware transformer using the patch attribute of transformer structure to encourage self-attention toward uncertain regions. Sun^31^ employed valuable and extra object-related edge semantics to force the learned representation learning to highlight object structure. Zhou^32^ connected edge features to the content features of each layer to enhance feature representation. Yue^33^ utilized initial region cues and boundary cues as input of area search module and area refinement module to enhance the extracted features. The paper proposes a saliency segmentation model that applies both coarse-to-fine structure and boundary guidance with edge-belts to further improve accuracy of heat maps of MVP.

**Figure 1:**
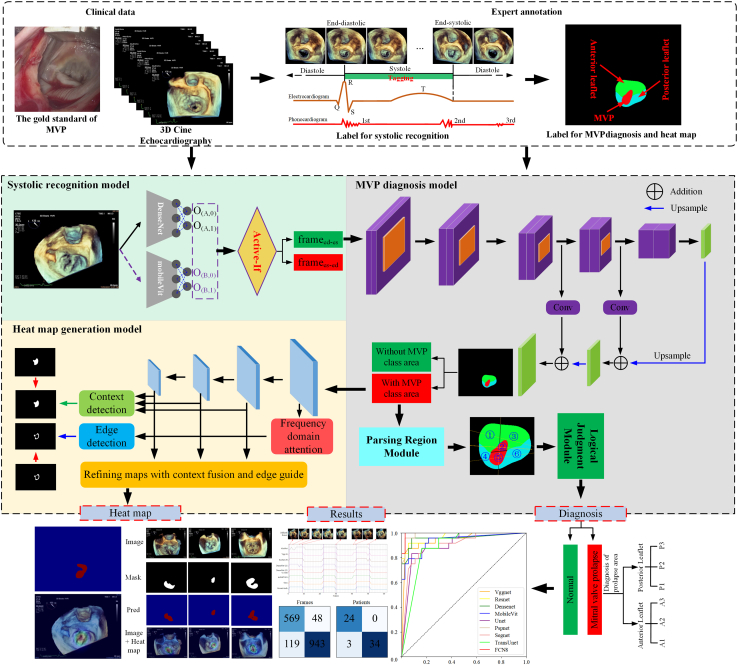
The overview of our artificial intelligence diagnosis and heatmap agent

The overview of our agent is shown in Figure 1. To train and validate our agent, we collect a certain amount of 3D cine echocardiography data. Ultrasound physicians manually annotate labels including frame flags of systolic frames and tissue region masks of AL, PL, and MVP. Our agent firstly identifies the frames of systolic by systolic recognition model. Then, MVP diagnosis model detects whether there are MVP regions in these systolic frames. If the proportion of systolic frames with MVP is greater than half, the case is diagnosed as MVP. And the frame with largest MVP area is selected, and the severe zone location of MVP is further parsed by parsing region module (PRM) and logical judement module (LJM). Next, the heat maps of systolic frames with MVP are obtained by heatmap generation model. Finally, we calculate results of these models with widely used evaluation metrics of classification and segmentation.

Compared to previous studies, our contributions can be summarized as follows: First, we develop an AI diagnosis and heatmap agent for MVP, which fills the gap of automatic diagnosis and visualization of MVP using 3D cine echocardiography. Second, we proposes a systolic recognition model, which integrates two deep learning networks and manages them through active-if module based on confidence level. Thirdly, we propose a saliency segmentation model whose boundary cue is guided by edge-belts with frequency domain attention mechanism to improve the effect of heatmap of MVP.

## Results

### Basic information and dataset

The total of 481 cardiac cycles (8422 frames) were collected from 151 subjects including 64 MVP patient studies and 87 control group studies to develop and validate the proposed agent. Only one video per patient is collected with 2–5 cardiac cycles. The basic information of MVP and control group study data are shown in Table 1.

**Table 1:** Basic information of MVP and control group study data

Parameter	Control group studies (*n* = 87, frame = 4681)	MVP studies (*n* = 64, frame = 3741)
Age, years	48.0 ± 10.6	49.4 ± 15.9
Gender, female (%)	54 (62.0%)	20 (31.3%)
Atrial fibrillation (%)	1 (1.1%)	15 (23.4%)
Patent foramen ovale (%)	51 (58.6%)	10 (15.6%)
Hypertension (%)	8 (9.2)	11 (17.2)
Myxomatous valve disease (%)	0	58 (90.6)
Endocarditis (%)	0	6 (9.4)
MR grade (N/M/Mod/Ms/S)	81/6/0/0/0	0/1/5/5/53
LAD (mm)	30.3 ± 4.4	42.4 ± 9.0
LVEDD (mm)	44.8 ± 4.5	55.1 ± 7.8
Mitral annular anteroposterior diameter (mm)	–	31.8 ± 5.8
Mitral annular lateromedial diameter (mm)	–	33.1 ± 6.2
LVEF (%)	67.1 ± 5.4	65.8 ± 7.1
E wave (m/s)	0.78 ± 0.18	1.35 ± 0.37
A wave (m/s)	0.62 (0.48 ± 0.75)	0.80 ± 0.23
E/A	1.3 ± 0.5	1.8 ± 0.7
Septal e’ wave velocity (cm/s)	10.2 ± 7.4	8.7 ± 2.4
Lateral e’ wave velocity (cm/s)	12.9 ± 3.2	11.8 ± 3.2
Average E/e’ ratio	7.4 ± 2.0	15.2 ± 6.7
The number of cardiac cycle	2–5	2–5
Spatial Size of 2D images (pixels)	800 × 600	800 × 600
Range of video length (frames)	26–156	34–123
Mean of video length (frames)	53.80	58.45

Data are represented as mean ± SD; MR, mitral regurgitation; N, None; M, mild; Mod, moderate; Ms, moderate-to -severe; S, Severe; LAD, left atrial diameter; LVEDD, left ventricular end diastolic diameter; LVEF, left ventricular ejection fraction; -, the characteristic was not assessed.

The number of videos, cardiac cycles, and images are described in Table 2 in detail. The systole periods of all cardiac cycles are labeled. These cine data are split with 55, 35, and 61 (the approximate ratio is 3:2:3) as the subset of training, validation, and testing, respectively. To evaluation of heatmap more reliably, it is excluded that low quality frames such as blurring and incomplete display which may result in inaccurate annotation. And 1326 frames (Train: 653, Val: 411, Test: 262) with clear prolapse area were annotated segmentation marks.

**Table 2:** Details of the dataset

Subset	Frame [Cardiac cycle/Video]			Frame of systole period			Frame of diastole period		
	Control	MVP	total	Control	MVP	total	Control	MVP	total
Train	1558 [93/30]	1526 [81/25]	3084 [174/55]	808	689	1497	750	837	1587
Val	1082 [63/20]	847 [45/15]	1929 [108/35]	562	413	975	520	434	954
Test	2041 [121/37]	1368 [78/24]	3409 [199/61]	1062	617	1679	979	751	1730
Total	4681 [277/87]	3741 [204/64]	8422 [481/151]	2432	1719	4151	2249	2022	4271

The segmentation annotation work was completed by four ultrasound physicians. Two senior physicians with over ten years of ultrasound experience independently annotated the training data (including the train and Val subset) and the test data (the test subset) respectively, under the guidance of ultrasound diagnostic reports and surgical notes. This aims to minimize individual biases and achieve unbiased evaluation as much as possible. These two physicians hold healthcare professional certificates with eight years of training program, received training in transesophageal echocardiography (TEE) interpretation, and completed more than 150 TEE examinations under supervision of director-level physician.

To ensure the high reliability of the annotations, two physicians conducted quality control checks on the annotations. In cases of discordance, the final decision is made by a senior physician with over fifteen years of ultrasound experience and extensive experience in intraoperative ultrasound. A junior physician pursuing master’s degree (resident physician) blindly annotated test subset for comparison with heatmap generation models.

The annotation work of phase of cardiac cycle was completed by a senior physician and quality control was implemented by two other senior physicians. The diagnosis of MVP is confirmed by surgery as the gold standard and does not require additional annotation.

In addition, to investigate the generalization of our agent on low-frequency (5–20 Hz) 3D echocardiography, another 60 cases (different from the subjects of the dataset in the main paper) of low-frequency echocardiography were collected as an external validation data. The evaluation experiments of our agent were conducted on the external validation set.

The external dataset we built contained 1367 images (281 cardiac cycles) from 60 3D transesophageal echocardiography videos with low frequency, including 30 videos from healthy studies and 30 videos from patient studies. Only one video per patient was collected. Most videos have three to four cardiac cycles (three cardiac cycles: 15 videos, and four cardiac cycles: 39 videos). The systolic periods of all cardiac cycles were labeled. All MVP studies’ ultrasound examinations were verified with the surgery. The number of videos, cardiac cycles, and images are described in Table 3 in detail.

**Table 3:** Details of the low-frequency external validation dataset

Frame [Cardiac cycle/Video]			Frame of systole period			Frame of diastole period		
health	patient	total	health	patient	total	health	patient	total
663 [112/30]	704 [106/30]	1367 [218/60]	361	352	713	302	352	654

### Model performance of systolic recognition model

**Figure 2:**
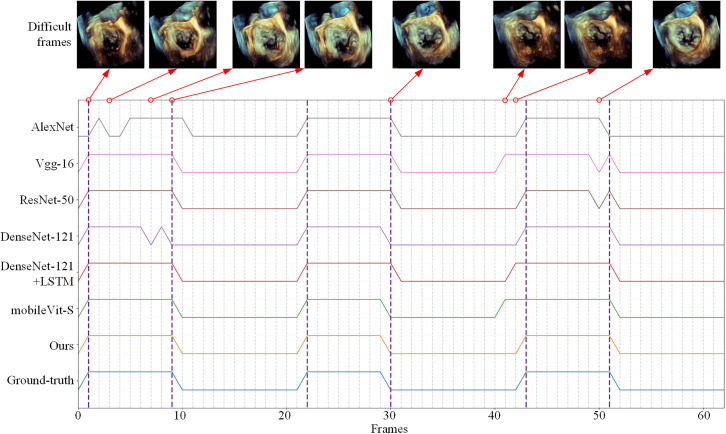
Visual comparisons of detection results of different models

Tables 4 and 5 display the evaluation results on frame level and cardiac-cycle level, respectively. The F1 and accuracy of our model are 95.78% and 95.80% with high precision on frame level. The accuracy of AccSys is as high as 97.07%. The average frame errors of end diastolic‌ (ED) and end systolic‌ (ES) are only 0.5427 and 0.0918. AE of ED and ES of our model is about 0.08/0.06 lower than that of best-second DenseNet-121+LSTM on cardiac-cycle level. An example of detection results of these models are displayed intuitively in Figure 2.

**Table 4:** Results of different models in frame level on SZH-3DEPD

Model	Pre↑	Rec↑	F1↑	Acc↑
AlexNet^34^	0.9389	0.9440	0.9414	0.9422
Vgg-16^35^	0.9472	0.9523	0.9498	0.9504
ResNet-50^36^	0.9423	0.9636	0.9528	0.9530
DenseNet-121^37^	0.9472	0.9624	0.9548	0.9551
DesNet-121+LSTM^38^	0.9378	0.9618	0.9497	0.9498
mobileVit-S^39^	0.9389	0.9702	0.9543	0.9542
Ours	0.9465	0.9696	0.9578	0.9580

**Table 5:** Results of different models in cardiac-cycle level on SZH-3DEPD

Model	AccSys↑	ED			ES		
		AE↓	E1↑	E2↑	AE↓	E1↑	E2↑
AlexNet	0.9209	0.6984	0.8592	0.9447	0.2142	0.9795	0.9948
Vgg-16	0.9252	0.6381	0.8643	0.9447	0.1428	0.9846	0.9948
ResNet-50	0.9660	0.6080	0.8542	0.9597	0.1071	0.9897	1.0
DenseNet-121	0.9431	0.5276	0.8793	0.9798	0.1275	0.9897	1.0
DesNet-121+LSTM	0.9754	0.6231	0.8592	0.9648	0.1581	0.9795	1.0
mobileVit-S	0.9476	0.5728	0.8693	0.9648	0.1020	1.0	1.0
Ours	0.9707	0.5427	0.8743	0.9748	0.0918	1.0	1.0

### Model performance of MVP diagnosis model

**Figure 3:**
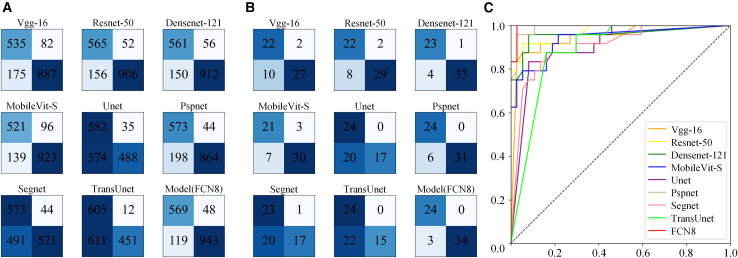
The confusion matrix of these results and ROC curves of MVP diagnosis (A) Confusion matrix of frame level. (B) Confusion matrix of patient level. (C) ROC curves of MVP diagnosis on the proportion of systolic frames with MVP.

As shown in Tables 6 and 7, segmentation model FCN8 obtains best results in MVP diagnosis in frame-levels and patient-levels. The accuracy of MVP in systolic frames can reach over 90%. The F1 of systolic frames of MVP patients is 87.20%. The Acc and F1 of MVP in patient-levels are 95.08% and 94.12% at the threshold 0.5 of the proportion of systolic frames with MVP. The AUC is 99.54% about threshold of proportion of systolic frames with MVP. The confusion matrix of these results and ROC curves of MVP diagnosis are listed in Figure 3. As shown in Table 8, the accuracy of SSZ_APL (Location of the most severe prolapse zone is either AL or PL) is 83.33%. The accuracy of DSZ_APL (whether there is MVP in AL and PL) is 77.08%. The accuracy of SSZ_SSZ (Location of the most severe prolapse zone is one of six sub zones, namely A1, A2, A3, P1, P2, and P3) is 70.83%.

**Table 6:** MVP diagnosis results of different models in frame-levels

Model	Pre↑	Rec↑	F1↑	Acc↑
Vgg-16	0.7535	0.8671	0.8063	0.8469
ResNet-50	0.7836	0.9157	0.8445	0.8761
DenseNet-121	0.7890	0.9092	0.8449	0.8773
mobileVit-S	0.7894	0.8444	0.8160	0.8600
Unet^40^	0.5035	0.9433	0.6565	0.6373
Pspnet^41^	0.7432	0.9287	0.8256	0.8559
Segnet^42^	0.5385	0.9287	0.6817	0.6814
TransUnet^43^	0.4975	0.9806	0.6601	0.6289
Model(FCN8)^44^	0.8270	0.9222	0.8720	0.9005

**Table 7:** MVP diagnosis results of different models in patient-levels

Model	Pre↑	Rec↑	F1↑	Acc↑	AUC↑
Vgg-16	0.6875	0.9167	0.7857	0.8033	0.9476
Resnet-50	0.7333	0.9167	0.8148	0.8361	0.9712
DenseNet-121	0.8519	0.9583	0.9020	0.9180	0.9571
MobileVit-S	0.7500	0.8750	0.8077	0.8361	0.9369
Unet	0.5455	1.0000	0.7059	0.6721	0.9053
Pspnet	0.8000	1.0000	0.8889	0.9016	0.9904
Segnet	0.5349	0.9583	0.6866	0.6557	0.9042
TransUnet	0.5217	1.0000	0.6857	0.6393	0.8856
Model(FCN8)	0.8889	1.0000	0.9412	0.9508	0.9954

**Table 8:** The results of main location of MVP of FCN8

Items	NOP	TP	Pre	Rec	F1	Acc
SSZ_APL	24	20	–	–	–	0.8333
DSZ_APL	–	–	0.8108	0.9375	0.8695	0.8125
SSZ_SSZ	24	17	–	–	–	0.7083

SSZ_APL, signal severe zone location of anterior and posterior leaflets; DSZ_APL, double severe zone of anterior and posterior leaflets; SSZ_SSZ, signal severe zone of six sub zones; NOP, number of patients.

### Model performance of heatmap generation model

**Figure 4:**
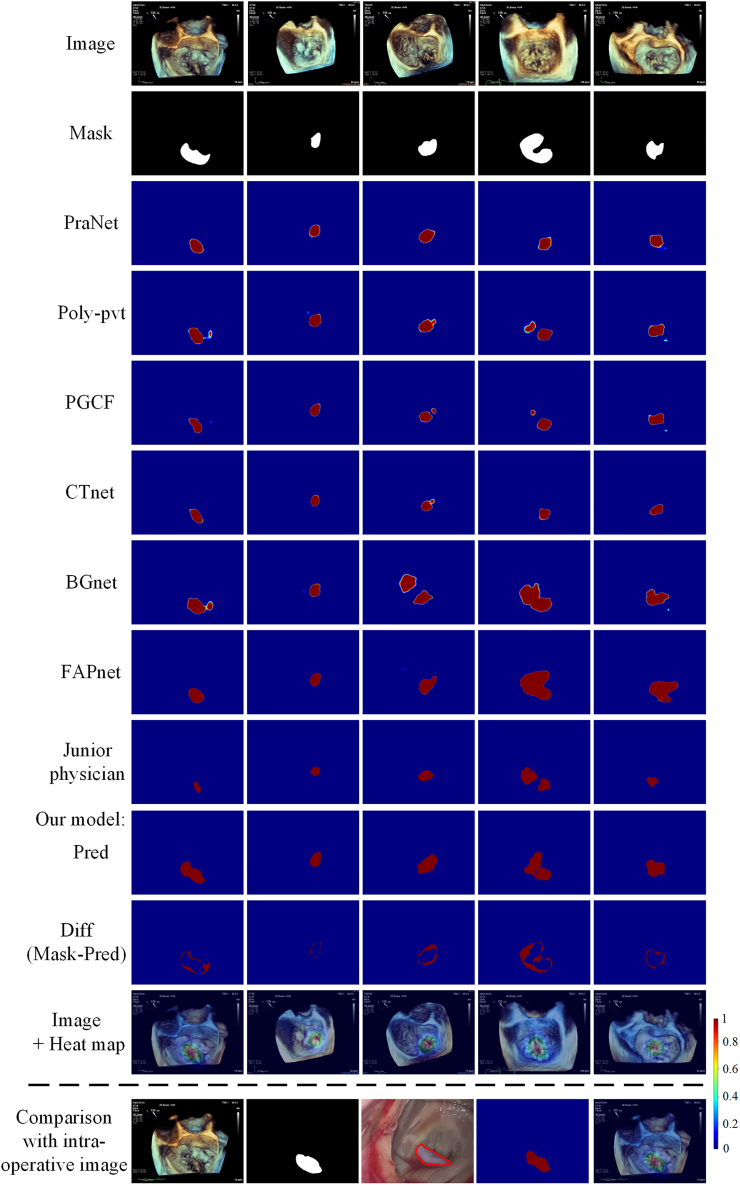
Examples of heat maps of different models and another physician

The average Dice between our proposed model and ground-truth is 70.35%, the average Iou is 56.39% as shown in Table 9. Compared to the manual annotation of a junior ultrasound physician, our proposed saliency segmentation model outperforms the manual annotation of the junior physician by approximately 5% on Iou (*p* = 0.0009) and Dice (*p* = 0.0001). The examples of heatmaps of different models and another physician are displayed in Figure 4.

**Table 9:** Iou and Dice of heat maps of different models and another physician

Model	Iou	Dice
PraNet	0.4925	0.6421
Poly-pvt	0.5130	0.6658
PGCF	0.4943	0.6407
CTnet	0.4568	0.6069
BGnet	0.5321	0.6752
FAPnet	0.5246	0.6678
Another Physician	0.5132	0.6475
Our model	0.5639	0.7035

### Model performance on 3D echocardiography with low-frequency

Our agent that is trained on SZH-3DEPD dataset with high-frequency data are evaluated on the external dataset with low-frequency data. The results of our agent are shown in Table 10. It can clearly see that our agent achieves high-accuracy phase detection at frame and cardiac-cycle level on low-frequency echocardiography. The F1 and accuracy of our model are 96.09% and 95.79% with high precision on frame level. The accuracy of AccSys is as high as 96.84%. Our agent has similar phase detection performance on 3D echocardiography at different frequencies.

**Table 10:** Results of our agent on the external dataset

EPD in frame level	Pre↑	Rec↑	F1↑	Acc↑			
	0.9727	0.9495	0.9609	0.9597			
EPD in cardiac-cycle level	AccSys↑	ED			ES		
		AE↓	E1↑	E2↑	AE↓	E1↑	E2↑
	0.9684	0.1651	0.9908	0.9908	0.1743	0.9862	0.9908
MVP diagnosis in frame level	Pre↑	Rec↑	F1↑	Acc↑			
	0.8641	0.9034	0.8833	0.8822			
MVP diagnosis in patient level	Pre↑	Rec↑	F1↑	Acc↑	AUC↑		
	0.8966	0.8667	0.8814	0.8833	0.9733		
Heatmap effects	Acc of qualified prediction (high-quality)						
	0.9034 (0.7892)						

**Figure 5:**
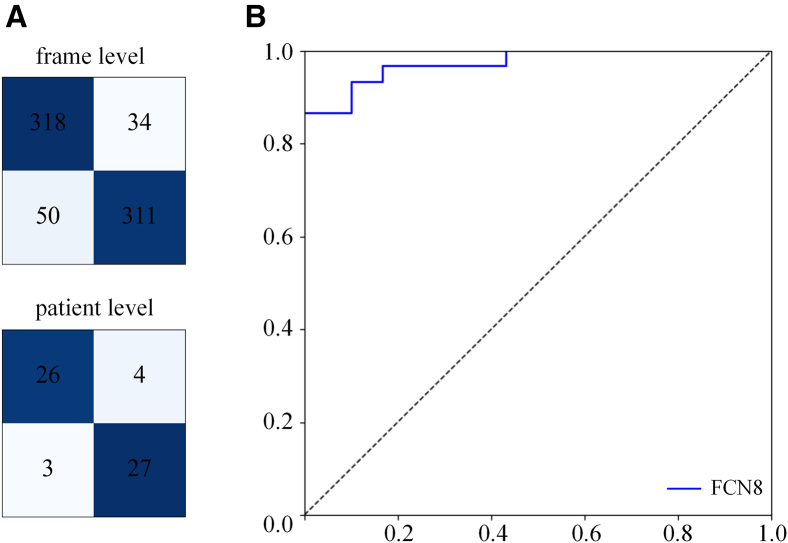
The confusion matrix of our agent and ROC curves of MVP diagnosis on the external dataset (A) Confusion matrix. (B) The ROC curve of MVP diagnosis on the proportion of systolic frames with MVP.

For the MVP diagnosis on low-frequency data, our agent obtains the MVP detection accuracy of 88.22% in systolic frames. The Acc and F1 of MVP in patient-levels are 88.33% and 88.14%. The AUC is 97.33% about threshold of proportion of systolic frames with MVP. The confusion matrix of these results and ROC curves of MVP diagnosis are listed in Figure 5. The MVP diagnostic performance of our agent shows the slight decrease on low-frequency echocardiography compared to high-frequency echocardiography, while its performance remains good with above 85% for all metrics.

**Figure 6:**
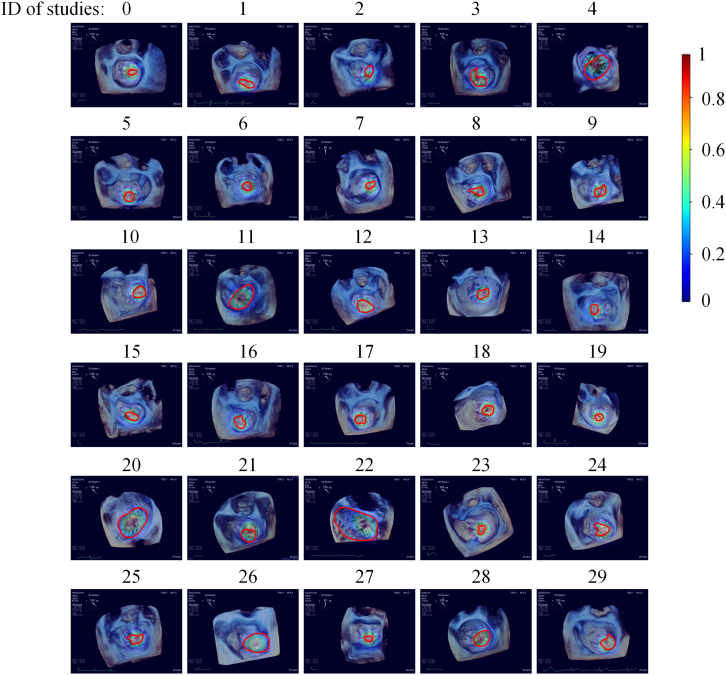
Examples of heatmap of one frame for each MVP study on the external dataset

For the heatmap of MVP, our agent generates the corresponding heat maps for all systolic frames of MVP studies. The Turing test is performed to evaluate the accuracy of qualified prediction based on heatmap prediction effects by a senior physician. The test results indicate that the accuracy of qualified prediction can reach over 90%, of which nearly 80% are high-quality predictions. The heatmap of one frame for each MVP study is shown in Figure 6. It can be intuitively seen that the heatmap predictions of our agent overall perform well.

Based on the previous analysis, our agent has good generalization on low-frequency 3D echocardiography.

## Discussion

In recent years, AI has been successfully applied to the automatic processing of 3D echocardiography, particularly with models based on deep learning. However, these models focus on measurement and reconstruction of cardiac structures based on 3D voxel echocardiography. Little attention has been paid to directly diagnosis and visualization based on 3D cine echocardiography. But 3D cine echocardiography is often used by physicians to diagnose MVP through observation and analysis in clinical practice. To address this gap, we develop an AI diagnosis agent with visualization of heatmap to assist clinical doctors in standardizing the diagnosis of MVP in this study. Our agent obtains 99.54% on AUC, 95.08% on Acc, and 94.12% on F1 for MVP diagnosis, which are highly consistent with the expert diagnosis results. The consistency of heat maps between our agent and senior physician is about 5% higher than that between junior and senior physicians on Iou and Dice. The AI agent contributes to improve the diagnosis accuracy in clinical practice, which provides a more efficient and accurate model for use by clinicians (especially inexperienced junior physicians) when diagnosing MVP.

High-precision systolic recognition is crucial as it is a prerequisite for MVP diagnosis and heatmap generation. The diagnosis of MVP is challenging due to the factors, such as the mitral annular nonplanarity during systole.^45^ MVP appears in the apical four-chamber view and is absent in roughly orthogonal long-axis views in most of these individuals. The diagnostic criteria of MVP were revised at the end of the 20th century, which indicates the difficulty of MVP diagnosis.^46^^,^^47^ 3D echocardiography clearly reconstructs the surface of the mitral valve and visually displays the location of MVP, which is highly beneficial for accuracy and reproducibility in the diagnosis and localization of MVP.^48^^,^^49^

We tested some existing deep learning models that extract single frame information based on CNN and transformer for systolic recognition. Then, the LSTM structure was added to DenseNet-121 model with best results to extract inter-frame information. It is found that inter-frame information can improve the accuracy of cardiac cycle AccSys, but it is not conducive to accurate recognition of ED/ES frames. The values of related indicators on frame error have increased. We adopted an integration strategy to improve detection accuracy by further extracting the output information of the classification models and constructing active-if module, which can steadily improve the performance of existing models (relevant experiments see section 1 of supplemental information).

Automatic diagnosis of MVP is essentially a classification task. However, we found that segmentation models can achieve higher accuracy than classification models as shown in Tables 6 and 7. Compared to classification models that learn in the manner of comparison, segmentation models can learn more accurate MVP features supervised by pixel-level labels. Some segmentation models exhibit significant overfitting, such as Unet and TransUnet. FCN8 preserves more shallow features through multiple upsampling and skip connections, which helps to finely reconstruct image information to achieve high segmentation accuracy. Simultaneously, segmentation models can obtain the specific location of MVP as shown in Figure S6 of supplemental information. This indicates segmentation models do not focus on other differences between data groups. Segmenting MVP for diagnose MVP makes the model have the ability of filtering out other interfering information (such as the impact of atrial fibrillation, PFO, mitral regurgitation, and the size of mitral valve itself) and achieve good robustness and reliability. On the other hand, segmentation models require additional pixel-level masks to be annotated by ultrasound physicians, which increase the difficulty of creating dataset and labor cost.

Generation of heatmap of MVP is saliency segmentation task. The main ultrasound display of MVP is continuous protrusion in spatial position during systole. Therefore the edge of MVP is lack of clear boundary. To alleviate the detection challenge, we proposed the heatmap generation model guided by edge-belts. Edge-belt guidance can not only enhance the learning of the edge of MVP, but also reduce sensitivity to bias of expert annotation, while making convergence easier for edge supervision. Edge-belt is detected based on the first layer features that contain more edge information. The content area of MVP is detected based on the remaining layer features. Besides, MVP causes abnormal deformation of the mitral valve, leading to changes in its frequency domain. Hence, our proposed model introduces learnable frequency domain attention mechanism, which is added to the feature extraction of first and second layers.

Good generalization of model is crucial for its application in the real world. On external low-frequency echocardiography dataset, the MVP diagnostic performance of our agent remains good with above 85% for all metrics as listed in Table 10. This indicates that our agent has good generalization ability on 3D echocardiography at different frequencies. The generalization of our agent in other aspects remains to be further studied and validated in future work, such as the improvement of our agent by domain adaptation techniques, and multi-center validation with diverse imaging protocols, equipment vendors, ethnic backgrounds, and age groups.

Our agent would provide standardizing diagnosis and intuitive visualization for the surgical management and treatment plans of MVP. However, due to the limitations of generalization research, and safety, etc., the AI agent could currently be used to assist trainees in diagnosing MVP by the prompt of heatmaps. This is confirmed to some extent by the comparison with the annotations of a junior physician. The drawback is that no robust reader-study of less-experienced readers is performed in our work.

The diagnosis and heatmap of the agent are intuitive and easy to understand. It can serve physicians with a simple explanation (The location where the diagnostic results are displayed and the sense of the color legend of heat maps). If trainees do not agree with the prediction of the agent, they should consult senior physicians or organize consultations to make a diagnosis and reduce misdiagnosis. It is ineffective for the detection of non-diagnostic MVP morphologies (NDM)^50^ and the early MVP without valve structural changes, as the prediction of the agent is based on the recognition of MVP areas.

### Conclusion

In this study, an artificial intelligence agent is proposed for diagnosis and heatmap of MVP. Two deep learning models are proposed to improve the accuracy of systolic recognition and heatmap segmentation, respectively. The AUC, accuracy, and F1 of MVP diagnosis are 99.54%, 95.08%, and 94.12% in patient-levels. The Dice and Iou of heatmap of MVP are 70.35% and 56.39%, which is about 5% higher than that of consistency between senior and junior physicians. The code of our agent: https://github.com/SHporcoRosso/mvp_agent.

### Limitations of the study

There are some limitations in this study. First, the establishment of the MVP database from a single research center, but multi-center large sample data would be more helpful to improve the accuracy of the machine learning. Second, this study only used ultrasound instruments from a single manufacturer to establish the dataset, which may mean the findings are not applicable to ultrasound instruments made by different manufacturers. Third, our validation results are based on surgical MVP data, so the performance on non-surgical mild MVP data (lack of gold standard-surgery findings) needs to be validated in the future. Finally, the AI agent predicts the location of sever zone of MVP with poor accuracy, which is an aspect we intend to address with the direction of our future research. The solution of this issue may require collecting more data as support, given its difficulty in identification of sub-zone of MVP.

## Resource availability

### Lead contact

Further information and requests for resources should be directed to and will be fulfilled by the lead contact, Yingying Liu (yingyingliu@ext.jnu.edu.cn).

### Materials availability

This study did not generate new unique reagents.

### Data and code availability

• The data are not publicly available due to hospital regulations. However, data requests with aims will be needed to assess the reasonability. After approval from the hospital and the corresponding authors, de-identified cine echocardiography data will be provided. Requests to access the datasets should be directed to corresponding author.
• Data are not publicly shared but is available upon reasonable request from the lead contact.
• The code of our agent is available at https://github.com/SHporcoRosso/mvp_agent. The code of labeling software is available at https://github.com/SHporcoRosso/Label-3DEPD.
• Any additional information required to reanalyze the data reported in this paper is available from the lead contact upon request.

## Acknowledgments

This work was supported by the 10.13039/501100002858China Postdoctoral Science Foundation (2022M12184); the 10.13039/501100001809National Natural Science Foundation of China (82102041); Project of Innovation of the Science and Technology Commission of Shenzhen City (JCYJ20210324113804013); Project of International cooperative research of the Science and Technology Commission of Shenzhen City (GJHZ20210705142205017 and GJHZ20210705142206019); Shenzhen People's Hospital Clinician Scientist Training Plan (SYWGSCGZH202302).

## Author contributions

D.Z.: investigation, methodology, software, validation, writing – original draft, funding acquisition; M.Y.: data curation, investigation, visualization, writing – review and editing; Xueyuan Lin: data curation, investigation, validation, writing – review and editing; Y.G.: data curation, visualization, writing – original draft; Xiaohua Liu. and Q.L.: data curation, validation, investigation, formal analysis; X.Z., Y.S., and S.L.: visualization, validation, data curation, formal analysis, writing – original draft; Y.H.: visualization, writing – original draft; L.C.: supervision, resources; J.X.: supervision, project administration; Xiaoxuan Lin.: conceptualization, software, investigation, writing – review and editing; Y.L.: conceptualization, project administration, funding acquisition, resources, writing – review and editing.

## Declaration of interests

The authors declare no competing interests.

## STAR★Methods

### Key resources table

**Table:** 

REAGENT or RESOURCE	SOURCE	IDENTIFIER
**Software and algorithms**		

Python	Van Rossum^51^	https://python.org/
PyTorch	Paszke et al.^52^	https://pytorch.org/
Opencv	Bradski et al.^53^	https://opencv.org/
Numpy	Harris et al.^54^	https://numpy.org/
Timm	Hugging Face^55^	https://github.com/huggingface/pytorch-image-models
Classification code for this paper	Weiaicunzai^56^	https://github.com/weiaicunzai/awesome-image-classification
Segmentation code for this paper	Meijiaxin^57^	https://github.com/taozh2017/Awesome-Polyp-Segmentation

### Experimental model and study participant details

The cine 3D cine echocardiography data used in this study originated from 151 subjects (64 MVP patient studies and 87 control group studies) undergoing TEE examination in the ultrasound department of Shenzhen People’s Hospital. This study was conducted in accordance with the Declaration of Helsinki (2013 revision) and approved by the ethics committee of Shenzhen People’s Hospital. The written informed consents were obtained from all studies prior to enrollment. This study did not divide the data based on gender and evaluate the influence of gender on the model.

All examinations had been performed using Epiq 7c echocardiographic machine (Philips Medical Systems, Andover, MA) and X7-2t transesophageal probes (frequency 2–7 MHz) with standard protocol and connected to the synchronous electrocardiogram. All imaging data incorporated in this study were obtained from conscious subjects undergoing pharyngeal mucosal anesthesia with topical 2% lidocaine gel during standardized image acquisition and archival procedures, and anesthetics or sedatives do not cause pharmacological interference on hemodynamic stability. The frame rate of 3DE was set previously. All 3D echocardiography images had been accomplished in 3D-zoom, single-beat mode, which provides the best spatial resolution. The mid-esophageal long-axis or two-chamber view is acquired.

The MVP is diagnosed by the parasternal long-axis window as systolic displacement of the mitral leaflet into the left ventricle of at least 2mm from the mitral annular plane in accordance with the established guidelines of the American Society of Echocardiography (ASE).^4^ All enrolled MVP studies’ ultrasound examinations were confirmed by surgery, which can prove the correctness of ultrasound diagnosis.

The control group comes from patients who are clinically highly suspected patent foramen ovale (PFO) with 3D echocardiography examination. A comprehensive set of well-defined exclusion criteria was established. The exclusion criteria were that the subjects have one or more the following conditions: moderate and severe valvular disease, heart failure, left ventricular ejection fraction less than 40%, coronary artery disease, congenital heart disease, Kawasaki disease, PFO combined with high-risk anatomical factors (such as those with concurrent atrial septal aneurysm, atrial septal defects, long tunnel-type PFO, Chiari network, long tunnel PFO (≥8mm), Eustachian valve, right-to-left shunt, excessive thickness of the septum secundum (≥10mm), or anatomical abnormalities secondary to aortic root dilation), and other cardiac sources of embolism such as thrombi, tumors, vegetation, history of cerebral infarction, lacunar infarction, obstructive sleep apnea, decompression sickness, and inability to acquire 3DE images.

### Method details

#### Development of systolic recognition model

Figure S1 shows the flowchart of the proposed systolic recognition model. In our model, two deep convolutional networks (DenseNet and mobileVit) are trained. One serves as main network A and the other serves as secondary network B. A is preferentially utilized. B is used to replace A only when confidence level of A is lower than the certain threshold and confidence level of B.

The pre-processing module for original images aims to extract region of interest (ROI) to improve data validity and data augmentation to increase data diversity. In our task, ROI is the imaging zone of 3D echocardiography, which are extracted by traditional threshold segmentation method. The data augmentation employs randomly cropping (400 × 400 to 224 × 224), horizontal flipping (probability: 0.5), rotation (range: −10 to 10°), and adjusting brightness, hue and contrast (range: 0.5 to 1.5 of original attribute value).

Active-if module is employed to determine whether network B replaces network A for prediction. Firstly, prediction positive category probability is obtained by performing exponential operation and normalization for outputs of network. Then, confidence level is defined as twice the difference between the prediction positive category probability and 0.5. This process can be formulated as

$$P_{\left(N,1\right)}=\frac{e^{O_{\left(N,1\right)}}}{e^{O_{\left(N,0\right)}}+e^{O_{\left(N,1\right)}}}$$

$${\text{Conf}}_{\;N\;}=2\times \left|P_{\left(N,1\right)}-0.5\right|$$

where O_(N, i)_ represents output of the i-th category of network N (A/B), and P_(N, i)_ represents prediction probability of the i-th category of network N. Conf _N_ is confidence level of network N. When activation conditions (Conf_A_ < C (The constant C is set to 0.4) and Conf_B_ > Conf_A_) are passed, network B is activated to predict the phase.

Table S1 shows the performance of our model with three different integration modules. Table S2 and Figure S3 explores the data volume requirements for our task. Figure S4 shows the interface of 3D EPD software we devolope to execute and test our model conveniently on other data.

#### Development of MVP diagnosis model

Figure S5 shows the flowchart of the MVP diagnosis model. The systolic frames firstly are fed into segmentation model FCN8. Segmentation maps are output about three categories, namely AL, PL and MVP. If the predicted segmentation map contains MVP area, the frame is considered to have MVP. For MVP diagnosis of study (one cine data), if the number of frames with MVP is greater than half of the systolic frames, the study is diagnosed as MVP patient. Then, the frame with the largest predicted MVP area is selected to predict the severe zone location of MVP through parsing region module and logical judgment module.

FCN8 is selected as the segmentation network, which preserves more shallow features through multiple upsampling and skip connections, which helps to finely reconstruct image information to achieve high segmentation accuracy. As shown in Tables 3 and 4, FCN8 obtains the best accuracy on MVP diagnosis in frame-levels and patient-levels. In addition, some auxiliary indicators (segmentation performance and the balance between the accuracy of MVP and no MVP frames) also display that FCN is the best segmentation model for our task (see Table S5 and Figure S6).

Tables S3 and S4 demonstrate the settings of two hyper-parameters, namely learning rate ratio K between the FCN8 parameters of two groups, and weights of classes. Table S6 displays the robustness of the model for atrial fibrillation.

#### Development of heat map generation model

Figure S7 displays the flowchart of the proposed heat map generation model. We firstly use the pyramid vision transformer (PVT-v2)^58^ to extract multi-layer features. Then, the coarse content map and edge map are generated by content detection module and edge detection module, respectively. Frequency domain attention module is employed to enhance the feature information of MVP. Next, content refinement module further extracts features of several layers (2^nd^ to 4^th^ layers) guided by the content map. Finally, the edge refinement module integrates multi-layer features in a progressive manner under the guidance of edge map to obtain refined saliency map.

Algorithm S1 displays the process of edge-belt generation method. Figures S8–S10 present the structure of each module of the model in detail. Figure S11 displays the heat maps of model assisting trainees in diagnosing MVP.

### Quantification and statistical analysis

For classification tasks (systolic recognition and MVP diagnosis), the performances of models are comprehensively evaluated with four widely used metrics: precision rate (Pre), Recall rate (Rec), and macroaveraging (F1) and Accuracy (Acc) on frame level. To comprehensively evaluate models, systolic recognition model is evaluated on cardiac-cycle level with seven metrics: accuracy of systole (AccSys), ED/ES frame error metrics of average error (AE), ratio of error 0 to 1 (E1), ratio of error 0 to 2 (E2). AccSys is used to evaluated in systolic recognition, which greatly improves the evaluation. AccSys = T/(P + N-T), where T represents the number of correctly predicted cardiac cycle. If any frame of systole period of cardiac cycle is detected, it is considered that the cardiac cycle is correctly predicted. P refers to the predicted number of cardiac cycle. The predicted number of systole period is considered as the number of detected cardiac cycle. N is the number of cardiac cycle of ground-truth, and the number of systole period of ground-truth is considered as the number of cardiac cycle of ground-truth. For severe zone location recognition of MVP, the metrics are the same as the metrics of frame level mentioned above, as only one frame is selected for each studies. For heat map of MVP (saliency segmentation task), we adopts Iou and Dice metrics widely used in segmentation tasks.

### Additional resources

This study did not use any type of experimental models. The study was conducted with the approval of the Medical Ethics Committee of Shenzhen People’s Hospital (reference number: LL-KY-202182). During the retrospective collection process, personal information that could identify individuals was eliminated. The informed consent was signed by all patients.

## Supplemental information

Document S1. Figures S1–S11, Tables S1–S6, Methods S1–S10 and Algorithm S1
