# Supplementary material for: Artificial intelligence diagnosis and heatmap agent for mitral valve prolapse using 3D cine echocardiography
Source: iScience. 2025 Jun 28;28(8):113033. doi: 10.1016/j.isci.2025.113033 (PMC12361798; doi:10.1016/j.isci.2025.113033)
Supplement: Document S1. Figures S1–S11, Tables S1–S6, Methods S1–S10 and Algorithm S1 [file mmc1.pdf]

iScience, Volume *28*

Supplemental information

Artificial intelligence diagnosis and heatmap agent for mitral valve prolapse using 3D cine echocardiography

Defu Zhang, Miaoru Yu, Xueyuan Lin, Ying Guo, Xiaohua Liu, Qian Liu, Xiaofang Zhong, Yuanyuan Sheng, Shuyu Luo, Yuxiang Huang, Lixin Chen, Jinfeng Xu, Xiaoxuan Lin, and Yingying Liu

1. The proposed systolic recognition model

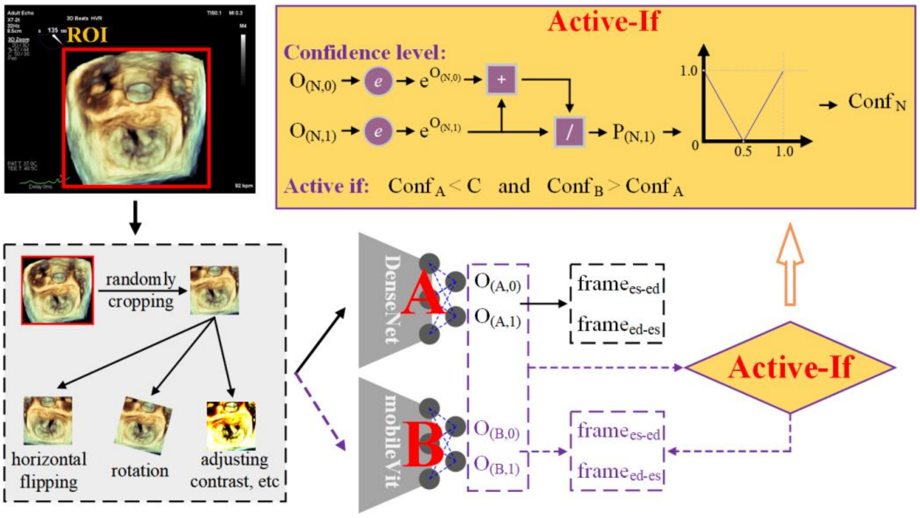

Figure S1. Architecture of our proposed systolic recognition model.

Table S1. Results of our model with different information integration modules in frame level

Integration modules	Pre↑	Rec↑	F1↑	Acc↑
Weighted-average	0.9454	0.9696	0.9573	0.9574
Active-voting	0.9454	0.9696	0.9573	0.9574
Active-if	0.9465	0.9696	0.9578	0.9580

Methods S1: The structure of information integration. It aims to integrate information from two deep learning networks to improve model performance. Three specific integration modules are designed, namely weighted-average module based on complementary theory, active-voting module based on voting mechanism, and active-voting module based on alternative mechanisms. The weighted-average module obtains the predicted vector by multiplying the prediction probability vectors of two networks with their confidence levels, and then summing them up to take the average. The active-voting module compares the confidence levels of two networks, and applies the output of the network with higher confidence level as the prediction vector of model. The predicted vectors of the two modules P_{avg} and P_{vot} can be expressed as :

$$P_{avg} = \frac{Conf_A \times P_A + Conf_B \times P_B}{2}$$

$$P_{vot} = \begin{cases} P_A & if \;\; Conf_A \geq Conf_B \\ P_B & other \end{cases}$$

where P_A and P_B represent the prediction probability vectors of the network A and network B respectively. $Conf_A$ and $Conf_B$ are the confidence level of two networks A and B. It can be clearly seen that weighted-average and active-voting modules make our model obtain the same performance.

The effect of active-if module on Rec is the same as that on the other two, which improves the model performance on Pre. Therefore, active-if module is adopted to integrate the information in our model.

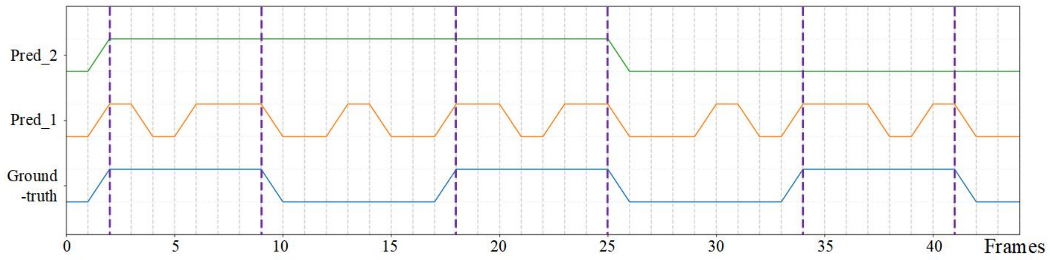

Figure S2. Example where frame error can not accurately evaluate method

Methods S2: The necessity of evaluating the accuracy of systole AccSys. The goal of our task is to accurately extract ED and ES frames. And it is definitely necessary to evaluate the frame error of ED/ES. However, why to evaluate the accuracy of systole AccSys? Because we notice that the frame error as shown in Figure S2 can not accurately evaluate the performance of method. Pred_1 accurately predicts all ED/ES frames, but in this case, the method should not be considered as a good method. Because if the systole period of one cardiac cycle is extracted, one of ED and ES is always wrong. Pred_2 is also similar. Although the extracted ED/ES are accurate, they belong to different cardiac cycle. And the extraction is meaningless. Therefore, evaluating AccSys is necessary for accurate evaluation method. Accurate recognition of cardiac cycle is the premise of accurate recognition of ED/ES frames. The AccSys of Pred_1 and Pred_2 are 0.375 and 0.333. And AccSys can accurately evaluate the performance of method in the case of Pred_1 and Pred_2.

Table S2. The Acc of DenseNet-121 with different data volumes

Train		Val		Total		Acc↑
Video	Image	Video	Image	Video	Image	
1	56	1	51	2	107	0.8424
2	106	2	120	4	226	0.8899
5	245	5	271	10	516	0.9193
15	760	10	525	25	1285	0.9284
25	1374	15	832	40	2206	0.9521
35	2027	20	1092	55	3119	0.9527
45	2619	25	1401	70	4020	0.9536
55	3084	35	1929	90	5013	0.9551

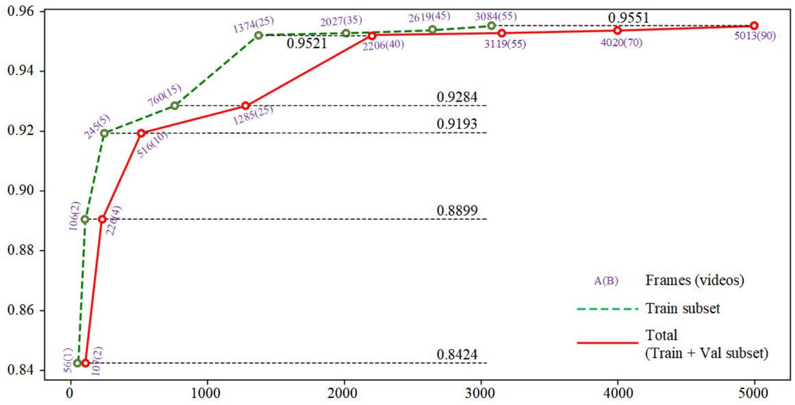

Figure S3. The curve of Acc with increasing data amount

Methods S3: The data demand for our task. It has become consensus that deep learning networks require a large amount of training data to optimize parameters. The datasets of nature images usually contain tens of thousands of images, and several large datasets collect hundreds of thousands or even tens of millions of images, such as ImageNet and COCO. However, do deep learning networks necessarily require tens of thousands of images to achieve good performance? How many data will basically reach the state of data saturation in our task? To clarify the above issues, a series of experiments are carried out with the network DenseNet-121 as data volume gradually increased.

The amount of data and corresponding result of Acc are listed in Table S2, and the curve of Acc with increasing data amount is displayed intuitively in Figure S3. It can be clearly seen that after the number of image exceeds 1374 (the number of videos exceeds 25), there is only negligible improvement of performance of DenseNet-121. This implies that our task reaches basically data saturation with about 2000 images (including about 800 of validation subset), and collecting videos from 40 to cases is sufficient for training data. Moreover, it also proves that not all tasks using deep learning networks require tens of thousands of data.

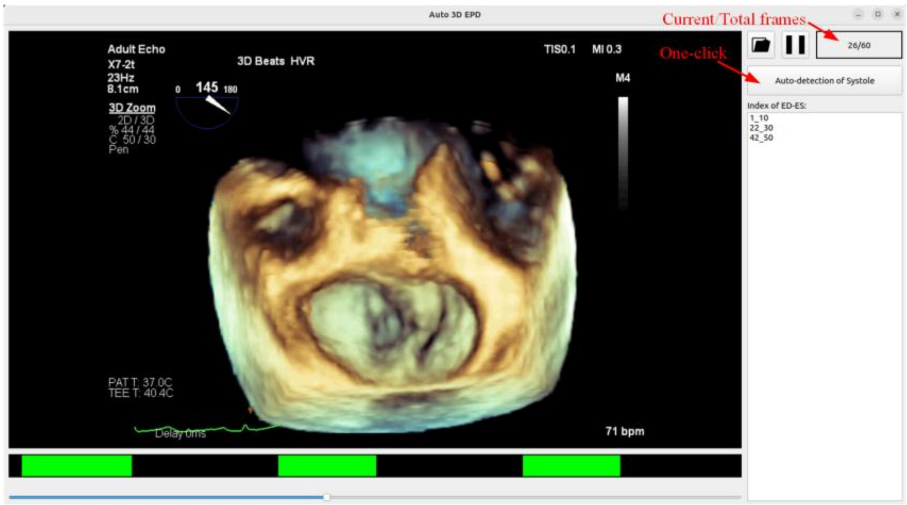

Figure S4. Interface of 3D EPD software

Methods S4: Interface of 3D EPD software. To execute and test our model conveniently on other data, we develop the software of 3D EPD as shown in Figure S4. The software supports one click detection of ED and ES. And Indexes of ED and ES are listed in text box on the right. And frames of systole are highlighted in corresponding position above progress bar. Current and total frames are displayed in the upper right corner. The code of the software and trained weights of our model on SZH-3DEPD are available at https://github.com/SHporcoRosso/3D-EPD.

2. The MVP diagnosis model

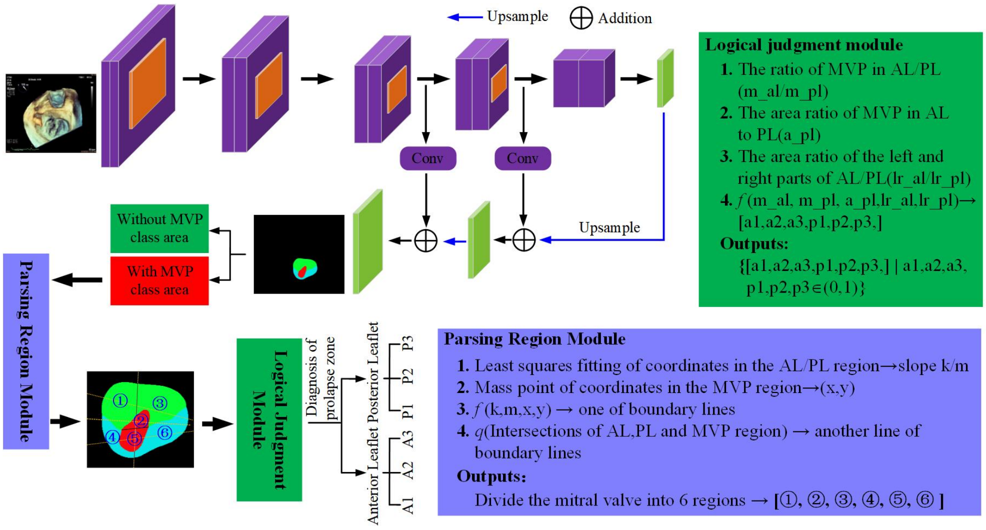

Figure S5. The flowchart of the MVP diagnosis model.

Table S3. MVP diagnosis results with different K values in frame-level

K	Pre↑	Rec↑	F1↑	Acc↑
1	0.6780	0.9692	0.7979	0.8195
5	0.7724	0.9238	0.8413	0.8719
10	0.8270	0.9222	0.8720	0.9005
20	0.8830	0.8687	0.8758	0.9095
30	0.9116	0.8185	0.8625	0.9041
40	0.8696	0.8541	0.8618	0.8993

Methods S5: The settings of two hyper-parameters, namely learning rate ratio K between the FCN8 parameters of two groups. Learning rate is initially set to 0.00001 for the parameters of Vgg-16 pre-trained on ImageNet (a large-scale public datasets) and 0.0001 for the parameters of the remaining modules, namely, the learning rate ratio K of the parameters of two groups is set to 10. A reasonable learning rate can reduce the oscillation phenomenon during training and facilitate convergence to the optimal solution. The parameters of Vgg-16 have been pre-trained on ImageNet, so a smaller learning rate is required. Similarly, the parameters of the remaining modules are randomly initialized, thus requiring a higher learning rate. Table S3 displays MVP diagnosis results with different values for learning rate ratio K of the parameters of two groups in frame-level. We can observe that when K is 20, F1 and Acc obtain the best results, but the difference from the result with k of 10 is very small. The Rec at K as 10 is significantly higher than that at K as 20. Overall, since we focus more on sensitivity, namely, Rec, the learning rate ratio K is set to 10.

Table S4. MVP diagnosis results with different weights of classes in frame-levels

Weights of classes (BG, AL, PL, MVP)	Pre↑	Rec↑	F1↑	Acc↑
[1,1,1,1]	0.8196	0.9060	0.8607	0.8922
[1,10,10,10]	0.8835	0.8849	0.8842	0.9148
[1,10,20,20]	0.8778	0.8963	0.8869	0.9160
[1,10,20,30]	0.8633	0.9109	0.8864	0.9142
[1,10,20,50]	0.8270	0.9222	0.8720	0.9005
[1,10,20,80]	0.8598	0.8947	0.8769	0.9077

Methods S5: The settings of the weights of classes. Appropriate weights of classes contribute to overcome the problem of class imbalance caused by different area sizes of various classes to improve the performance for segmentation networks. In our task, there is a situation that the background area is large and MVP area is small. A series of experiments with different weights of classes are conducted. As shown in Table S4, the network performance can always be improve by adding certain weights based on the size of the category area. We focus more on sensitivity, namely, Rec, so weights of classes are setting as 1, 10, 20, 50 for background (BG), AL, PL and MVP to obtain the highest Rec.

Table S5. Results of different segmentation networks

Segmentation network	MVP-area		Acc of frames	
	Iou	Dice	MVP	No-MVP
Unet	0.2841	0.4002	0.9433	0.4595
Pspnet	0.4623	0.6027	0.9287	0.8136
Segnet	0.3531	0.4741	0.9287	0.5377
TransUnet	0.3265	0.4680	0.9806	0.4247
Model(FCN8)	0.5035	0.6277	0.9222	0.8879

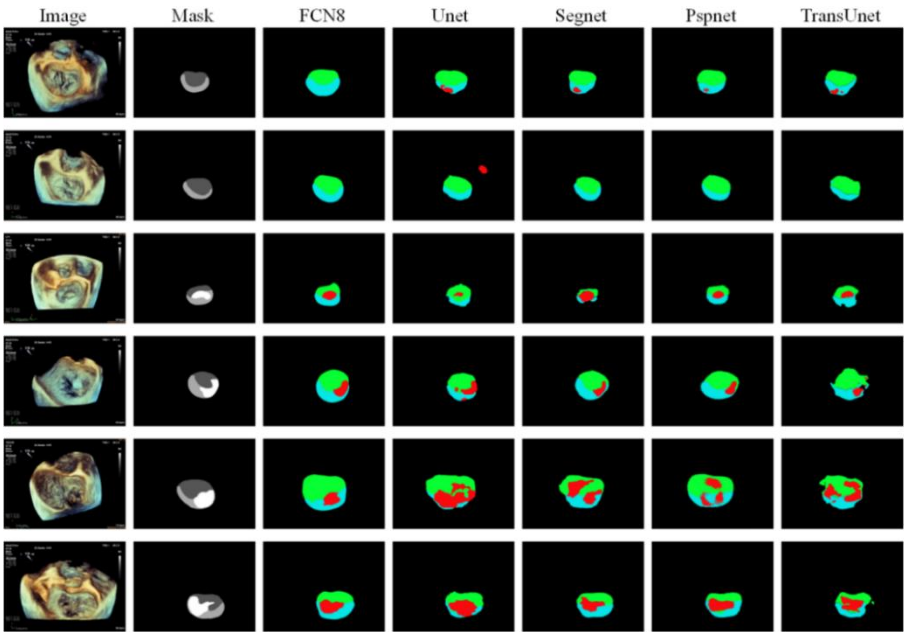

Figure S6. Examples of segmentation maps of different segmentation networks

Methods S6: The selection of segmentation network. FCN8 obtains the best results in both IoU and Dice. Although Unet and TransUnet have higher Acc than FCN8 in the MVP frames, they have very low ACC in the No-MVP frames. Figure S6 shows several segmentation maps of these segmentation networks. It can be intuitively seen that FCN8 has the best segmentation effect. Based on the above analysis, FCN8 is selected as the segmentation network.

Table S6. MVP diagnosis results of FCN8 without AFib study data

Testing data		Pre↑	Rec↑	F1↑	Acc↑	AUC↑
frame	Without AFib	0.7871	0.9263	0.8511	0.8998	-
level	With AFib	0.8270	0.9222	0.8720	0.9005	-
patient	Without AFib	0.8500	1.0000	0.9189	0.9444	0.9952
level	With AFib	0.8889	1.0000	0.9412	0.9508	0.9954

Methods S7: The robustness of the model for atrial fibrillation. To quantitatively evaluate the robustness of the model for atrial fibrillation study data, all atrial fibrillation (AFib) study data of the test set are removed, and the diagnostic results of the model FCN8 are evaluated. As shown in Table S6, it is evident that AFib data does not have a significant impact on model performance, especially in terms of comprehensive indicators. FCN8 has good robustness on the AFib data. This also demonstrates the superiority of the segmentation model for MVP diagnosis, as it only focuses on the recognition of MVP area and suppresses interference from other information such as AFib.

3. The proposed heat map generation model

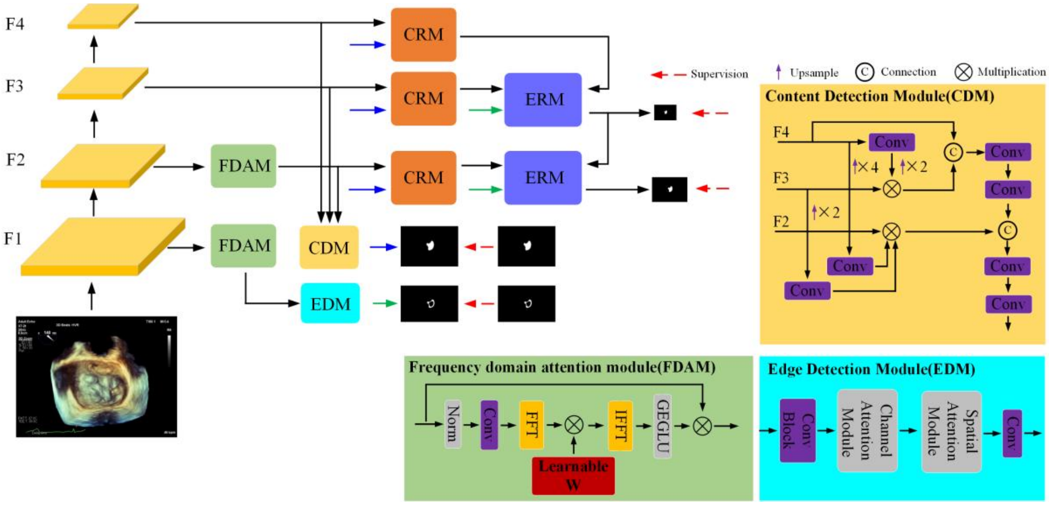

Figure S7. The flowchart of the proposed method.

**Algorithm S1** the process of edge-belt generation method
**Input:** mask M
k←0.62
n_area ← num_nonzero(M)
k_e ← sqrt(n_area)*k
$M_p \leftarrow Avg\_pool(\mathrm{M}, \mathrm{ksize} = \mathrm{k\_e})$
$M_g \leftarrow Abs(M_p, \mathrm{M}) \otimes \mathrm{M}$
$M_e \leftarrow Bin(M_g)$
**Output:** M_e

Methods S8: The edge-belt generation method. The goal of method is that edge-belt with appropriate width is adaptively generated based on the area of MVP. The detailed process is shown in Algorithm S1. Firstly, the average pooling kernel is determined based on the area of MVP and proportion coefficient k. Then, The $\mathrm{M_p}$ (M after being pooled) performs absolute value operation and dot-product operation with M to obtain edge grayscale map $\mathrm{M_g}$. Finally, the final edge-belt map $\mathrm{M_e}$ is obtained by Binarization of $\mathrm{M_g}$.

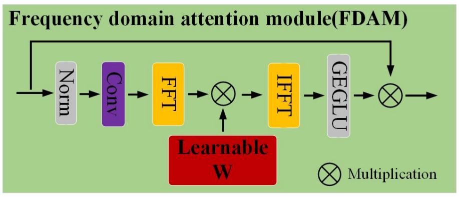

Figure S8. The frequency domain attention module.

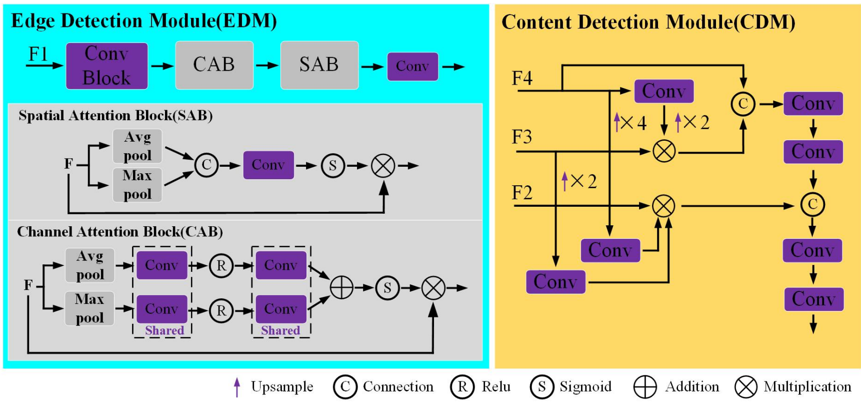

Figure S9. The edge detection and content detection modules.

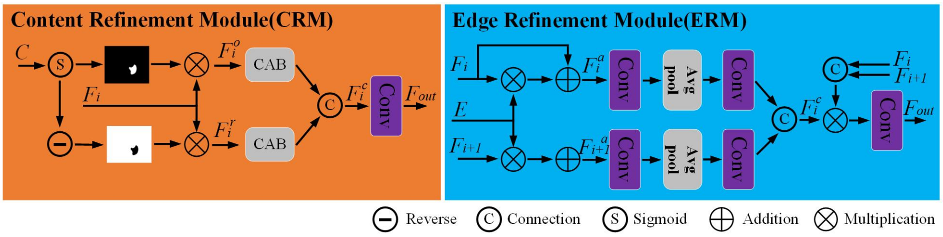

Figure S10. The content refinement and edge refinement modules.

Methods S9: The structure of each module of the model.

Frequency domain attention module (FDAM) is employed to enhance the feature information of MVP. As shown in Figure S8, firstly, features X are processed by a layer normalization and a 1×1 convolution. Then, similar to the JPEG compression algorithm, features are converted into frequency domain representation using fast fourier transform (FFT). A learnable matrix W is setting to different frequencies with different weights, which can adaptively determine which frequency information should be preserved. Next, Frequency domain features are converted back into the spatial domain by inverse fast fourier transform (IFFT). a GEGLU function regularizes the features. The above process can be formulated by $X_o = G(F^{-1}(W(F(\mathrm{Conv}(x)))))$ where F , F^{-1} and G denote FFT, IFFT and GEGLU function. Finally, The frequency domain attention features X_{att} is obtain by a

dot-product operation with X and X_o .

The edge detection module (EDM) generates edge-belt maps with a series of serial operations including convolution block, channel attention block (CAB) and spatial attention block (SAB) as shown in Figure S9, which can be represented as $F_{out} = Conv(CAB(SAB(Conv(Conv(Conv(F_1))))))$. Specifically, CAB consists of channel-wise operation (mean $AP(\bullet)$ and maximum $MP(\bullet)$), convolution layer $Conv(\bullet)$, sigmoid function $S(\bullet)$, add-wise operation $\oplus$ and dot-product operation $\otimes$. The process can be expressed as $F_c = F \otimes S(Conv(R(Conv(AP(F)))) \oplus Conv(R(Conv(AP(F)))))$. Similarly, SAB consists of spatial-wise operation (mean $AP(\bullet)$ and maximum $MP(\bullet)$), convolution layer $Conv(\bullet)$, sigmoid function $S(\bullet)$, connection operation © and dot-product operation $\otimes$ .The process can be expressed as $F_S = F \otimes S(Conv(AP(F) © MP(F)))$.

The content detection module(CDM) adopts multi-layer fusion approach to integrate the features of different layers with convolution layer $Conv(\bullet)$, dot-product operation $\otimes$ and connection operation ©. The operations can be expressed as

$$F_{3m} = (Conv(F_4) \otimes F_3) © F_4$$

$$F_{2m} = (Conv(F_4) \otimes Conv(F_3) \otimes Conv(F_2)$$

$$F_{out} = Conv(Conv(Conv(Conv(F_{3m})) © F_{2m}))$$

To refine content maps, we set content refinement module (CRM) with content map C and F_i as inputs. Specifically, F_i is firstly multiplied by C and $1-C$ in an element-wise manner respectively to obtain F_i^o and F_i^r . Then, as shown in Figure S10, F_i^c connect F_i^o and F_i^r after being processed by CAB operation. Finally, F_{out} is get by a 1×1 convolution operation for F_i^c .

The edge refinement module (ERM) aims to further refine edge regions, which tasks F_i , F_{i+1} and edge map E as inputs. As shown in Fig.5, F_i and F_{i+1} firstly multiplies E , and then add-wise operation with themself to obtain F_i^a and F_{i+1}^a . After that, the resulting feature maps are processed by a convolution layer, a global average pooling, and a convolution layer sequentially. Then the features of two branches are connected together to obtain F_i^c . Finally, F_{out} is obtained by a

pot-product operation between F_i^c and the connection features of F_i and F_{i+1} , a convolution layer.

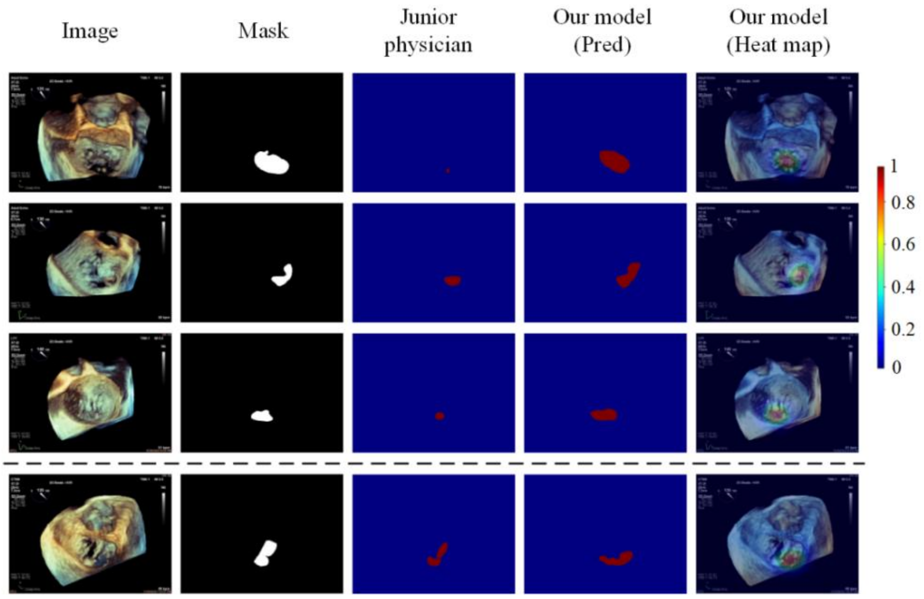

Figure S11. Several examples of heat maps of the model warning trainees.

Methods S10: The heat maps of model assisting trainees in diagnosing MVP. Our heat map generation model outperforms the manual annotation of a trainees by approximately 5% on Dice and Iou. To visually demonstrate the auxiliary effect of the model for trainees in diagnosing MVP, Figure S11 displays several examples of heat maps of the model warning trainees of misdiagnosis. It can be clearly seen that the trainees makes significant errors in judging the MVP area in the examples at the first three lines, which can lead to misdiagnosis of the severity of MVP and even missed diagnosis. The last line example shows the situation where the predicted heat map is worse than that of trainees. If trainees do not agree with the model prediction, they could consult senior physicians or organize consultations to make diagnosis. The heat map prompts are helpful in assisting and warning trainees to potential errors in identifying MVP areas and reducing misdiagnosis.
